# Supplementary material for: HIVprotI: an integrated web based platform for prediction and design of HIV proteins inhibitors
Source: J Cheminform. 2018 Mar 9;10:12. doi: 10.1186/s13321-018-0266-y (PMC5845081; doi:10.1186/s13321-018-0266-y)
Supplement: Supplementary file 2 — Additional file 2. Source code of HIVProtI web server. [file 13321_2018_266_MOESM2_ESM.zip › HIVprotI_Source-code/hivprotI/weka-3-6-6/doc/weka/core/Javadoc.html]

Javadoc


---


|  |  |  |
| --- | --- | --- |
| **Overview** \| **Package** \| **Class** \| **Tree** \| **Deprecated** \| **Index** \| **Help** \| **Weka's home** | |  |
| **PREV CLASS**   **NEXT CLASS** | **FRAMES**    **NO FRAMES**     **All Classes** |  |
| SUMMARY: NESTED \| FIELD \| CONSTR \| METHOD | DETAIL: FIELD \| CONSTR \| METHOD |  |


---


## weka.core Class Javadoc

```
java.lang.Object
  weka.core.Javadoc
```

**All Implemented Interfaces:**: OptionHandler, RevisionHandler

**Direct Known Subclasses:**: AllJavadoc, GlobalInfoJavadoc, OptionHandlerJavadoc, TechnicalInformationHandlerJavadoc

---

``` public abstract class Javadoc extends java.lang.Object implements OptionHandler, RevisionHandler ```

Abstract superclass for classes that generate Javadoc comments and replace
the content between certain comment tags.

**Version:**
:   $Revision: 1.6 $

**Author:**
:   fracpete (fracpete at waikato dot ac dot nz)

---

**Constructor Summary**

`Javadoc()`


| **Method Summary** | |
| --- | --- |
| `java.lang.String` | `generate()`             generates either the plain Javadoc (if no filename specified) or the updated file (if a filename is specified). |
| `java.lang.String` | `generateHelp()`             generates a string to print as help on the console |
| `java.lang.String` | `getClassname()`             returns the current classname |
| `java.lang.String` | `getDir()`             returns the current dir containing the class to update. |
| `java.lang.String[]` | `getOptions()`             Gets the current settings of this object. |
| `boolean` | `getSilent()`             whether output in the console is suppressed |
| `boolean` | `getUseStars()`             whether the Javadoc is prefixed with "\*" |
| `java.util.Enumeration` | `listOptions()`             Returns an enumeration describing the available options. |
| `void` | `setClassname(java.lang.String value)`             sets the classname of the class to generate the Javadoc for |
| `void` | `setDir(java.lang.String value)`             sets the dir containing the file that is to be updated. |
| `void` | `setOptions(java.lang.String[] options)`             Parses a given list of options. |
| `void` | `setSilent(boolean value)`             sets whether to suppress output in the console |
| `void` | `setUseStars(boolean value)`             sets whether to prefix the Javadoc with "\*" |
| `java.lang.String` | `updateJavadoc()`             generates the Javadoc and returns it applied to the source file if one was provided, otherwise an empty string. |

**Methods inherited from class java.lang.Object**

`equals, getClass, hashCode, notify, notifyAll, toString, wait, wait, wait`

**Methods inherited from interface weka.core.RevisionHandler**

`getRevision`

**Constructor Detail**

### Javadoc

```
public Javadoc()
```


**Method Detail**

### listOptions

```
public java.util.Enumeration listOptions()
```

:   Returns an enumeration describing the available options.

    :   **Specified by:**: `listOptions` in interface `OptionHandler`
    :   **Returns:**: an enumeration of all the available options.

---


### setOptions

```
public void setOptions(java.lang.String[] options)
                throws java.lang.Exception
```

:   Parses a given list of options.

    :   **Specified by:**: `setOptions` in interface `OptionHandler`
    :   **Parameters:**: `options` - the list of options as an array of strings **Throws:**: `java.lang.Exception` - if an option is not supported

---


### getOptions

```
public java.lang.String[] getOptions()
```

:   Gets the current settings of this object.

    :   **Specified by:**: `getOptions` in interface `OptionHandler`
    :   **Returns:**: an array of strings suitable for passing to setOptions

---


### setClassname

```
public void setClassname(java.lang.String value)
```

:   sets the classname of the class to generate the Javadoc for

    :   **Parameters:**: `value` - the new classname

---


### getClassname

```
public java.lang.String getClassname()
```

:   returns the current classname

    :   **Returns:**: the current classname

---


### setUseStars

```
public void setUseStars(boolean value)
```

:   sets whether to prefix the Javadoc with "\*"

    :   **Parameters:**: `value` - true if stars are used

---


### getUseStars

```
public boolean getUseStars()
```

:   whether the Javadoc is prefixed with "\*"

    :   **Returns:**: whether stars are used

---


### setDir

```
public void setDir(java.lang.String value)
```

:   sets the dir containing the file that is to be updated. It is the dir
    above the package hierarchy of the class.

    :   **Parameters:**: `value` - the directory containing the classes

---


### getDir

```
public java.lang.String getDir()
```

:   returns the current dir containing the class to update. It is the dir
    above the package name of the class.

    :   **Returns:**: the current directory

---


### setSilent

```
public void setSilent(boolean value)
```

:   sets whether to suppress output in the console

    :   **Parameters:**: `value` - true if output is to be suppressed

---


### getSilent

```
public boolean getSilent()
```

:   whether output in the console is suppressed

    :   **Returns:**: true if output is suppressed

---


### updateJavadoc

```
public java.lang.String updateJavadoc()
                               throws java.lang.Exception
```

:   generates the Javadoc and returns it applied to the source file if one
    was provided, otherwise an empty string.

    :   **Returns:**: the generated Javadoc **Throws:**: `java.lang.Exception` - in case the generation fails

---


### generate

```
public java.lang.String generate()
                          throws java.lang.Exception
```

:   generates either the plain Javadoc (if no filename specified) or the
    updated file (if a filename is specified). The start and end tag for
    the global info have to be specified in the file in the latter case.

    :   **Returns:**: either the plain Javadoc or the modified file **Throws:**: `java.lang.Exception` - in case the generation fails

---


### generateHelp

```
public java.lang.String generateHelp()
```

:   generates a string to print as help on the console

    :   **Returns:**: the generated help


---


|  |  |  |
| --- | --- | --- |
| **Overview** \| **Package** \| **Class** \| **Tree** \| **Deprecated** \| **Index** \| **Help** \| **Weka's home** | |  |
| **PREV CLASS**   **NEXT CLASS** | **FRAMES**    **NO FRAMES**     **All Classes** |  |
| SUMMARY: NESTED \| FIELD \| CONSTR \| METHOD | DETAIL: FIELD \| CONSTR \| METHOD |  |


---
